# Supplementary material for: Distributed genetic architecture across the hippocampal formation implies common neuropathology across brain disorders
Source: Nat Commun. 2022 Jun 15;13:3436. doi: 10.1038/s41467-022-31086-w (PMC9200849; doi:10.1038/s41467-022-31086-w)
Supplement: Supplementary file 1 — Supplementary Information [file 41467_2022_31086_MOESM1_ESM.pdf]

Supplementary Information

Distributed genetic architecture across the hippocampal formation implies common neuropathology across brain disorders

Shahram Bahrami[1], Kaja Nordengen[1,2], Alexey A. Shadrin[1,3], Oleksandr Frei[1], Dennis van der Meer[1,4], Anders M. Dale[5,6,7], Lars T. Westlye[1,3,8], Ole A. Andreassen[1,3], Tobias Kaufmann[1,9]

1 Norwegian Centre for Mental Disorders Research, Division of Mental Health and Addiction, Oslo University Hospital & Institute of Clinical Medicine, University of Oslo, Oslo, Norway
2 Department of Neurology, Oslo University Hospital, Oslo, Norway
3 KG Jebsen Centre for Neurodevelopmental Disorders, University of Oslo, Oslo, Norway
4 School of Mental Health and Neuroscience, Faculty of Health, Medicine and Life Sciences, Maastricht University, Maastricht, The Netherlands
5 Department of Radiology, School of Medicine, University of California, San Diego, CA, USA
6 Department of Neurosciences, University of California San Diego, La Jolla, CA 92037, USA
7 Center for Multimodal Imaging and Genetics, University of California at San Diego, La Jolla, CA, 92037, USA
8 Department of Psychology, University of Oslo, Oslo, Norway
9 Department of Psychiatry and Psychotherapy, Tübingen Center for Mental Health, University of Tübingen, Tübingen, Germany

Table of content

Supplementary item Page

Supplementary Figure 1 ………………… 2

Supplementary Figure 2 ………………… 3

Supplementary Figure 3 ………………… 4

Supplementary Figure 4 ………………… 5

Supplementary Figure 5 ………………… 6

Supplementary Figure 6 ………………… 7

Supplementary Figure 7 ………………… 8

Supplementary Figure 8 ………………… 9

Supplementary Figure 9 ………………… 10

Supplementary References ……………… 11

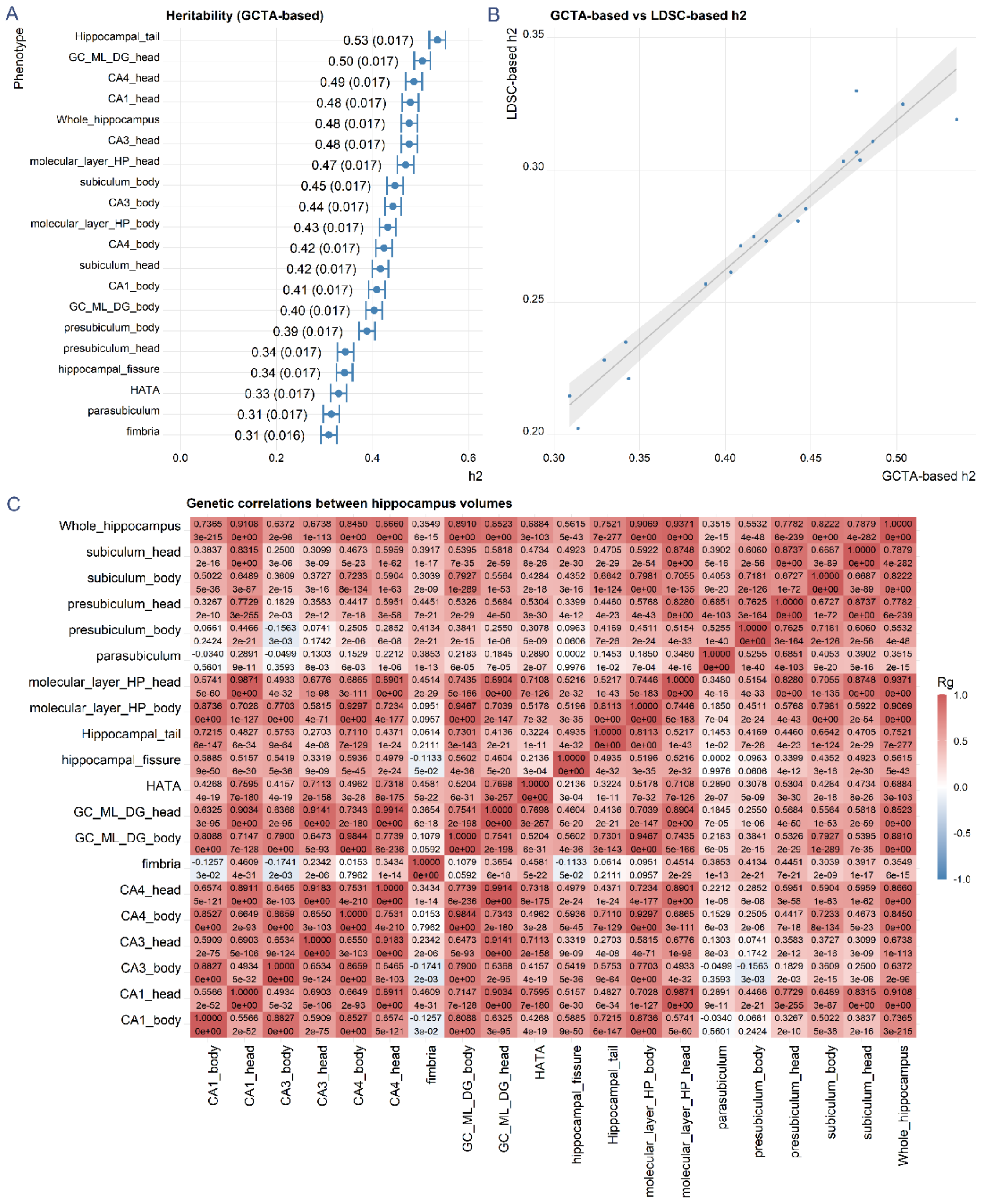

Supplementary Figure 1. Heritability of hippocampus volumes and their genetic correlation from univariate GWAS. GCTA=Genome-wide Complex Trait Analysis, LDSC=Linkage Disequilibrium Score. (A) GCTA-based heritability estimates (n=35,411). The numbers depict heritability estimates (h2), with standard error in brackets. Error bars reflect standard error. (B) Comparison of GCTA-based and LDSC-based heritability. As expected, GCTA-based estimates are generally higher, yet both methods show similar patterns (Pearson correlation between the two: r=0.97, P=4e-13). The shaded error band reflects confidence intervals. (C) LD-score regression based genetic correlations (Rg) between each pair of regions, using the univariate GWAS summary statistics. P-values are two-tailed. The overall lowest genetic correlations were with fimbria, which is also the structure with lowest heritability. As an additional sanity check we also calculated the genetic correlations between our 20 univariate GWAS summary statistics and the summary statistic of the whole hippocampus from a previous GWAS[1] and found that all volumes were significantly associated (smallest Rg was with fimbria: Rg=.40, P=6e-07).

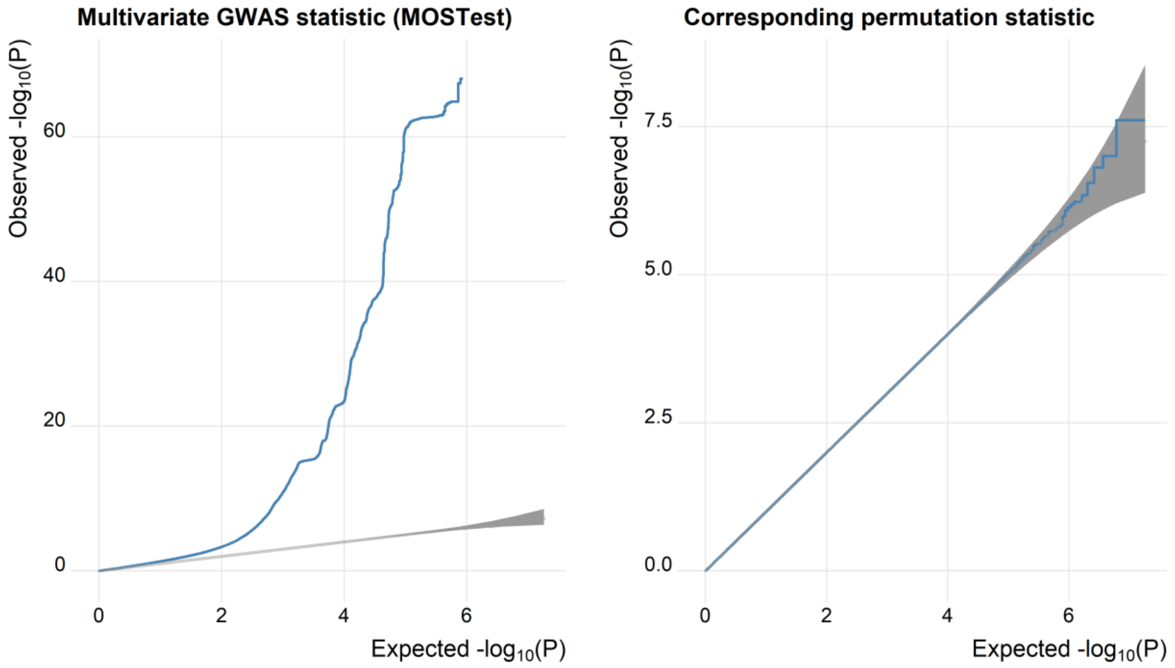

Supplementary Figure 2. Quantile-quantile plots from MOSTest analysis. The left panel depicts signal from MOSTest analysis. The right panel shows test statistics under null (from permutation testing) and confirms validity of the MOSTest test statistics.

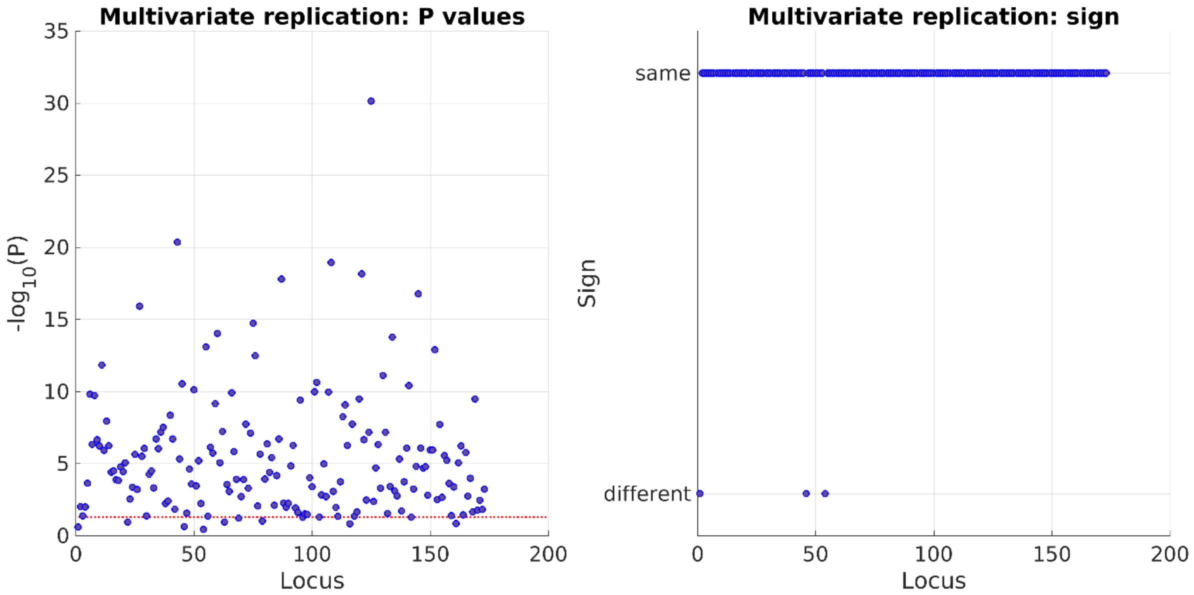

Supplementary Figure 3. Multivariate replication analysis using independent data from 5262 individuals with non-white ethnicity. Using a multivariate replication procedure[2] (see *Methods*), we found that 68% of the loci replicated at P<.05 and 98% showed the same effect direction. P-values are denoted as $-\log_{10}(P)$.

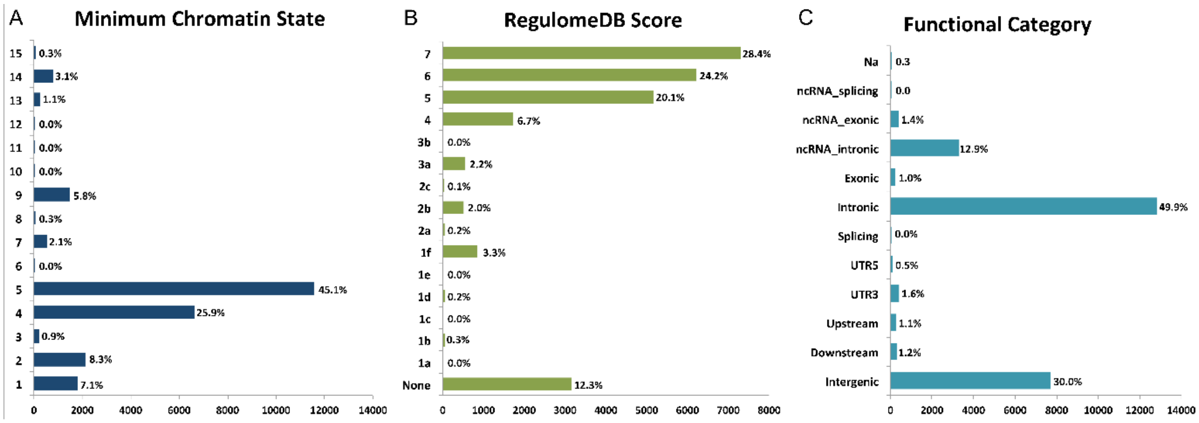

Supplementary Figure 4. Distribution of the annotation for all SNPs in the significant genetic loci from the hippocampus GWAS including (A) the minimum chromatin state across 127 tissue and cell types for SNPs in the significant genomic loci, with lower states indicating higher accessibility and states 1–7 referring to open chromatin states, (B) the distribution of RegulomeDB scores for SNPs in the significant genomic loci, with a low score indicating a higher likelihood of having a regulatory function and (C) the distribution of functional consequences of SNPs in the significant genomic risk loci. The chromatin states are 1=Active Transcription Start Site (TSS); 2=Flanking Active TSS; 3=Transcription at gene 5' and 3'; 4=Strong transcription; 5=Weak Transcription; 6=Genic enhancers; 7=Enhancers; 8=Zinc finger genes & repeats; 9=Heterochromatic; 10=Bivalent/Poised TSS; 11=Flanking Bivalent/Poised TSS/Enh; 12=Bivalent Enhancer; 13=Repressed PolyComb; 14=Weak Repressed PolyComb; 15=Quiescent/Low. RegulomeDB categories reflect: 1a: eQTL + TF binding + matched TF motif + matched DNase Footprint + DNase peak; 1b: eQTL + TF binding + any motif + DNase Footprint + DNase peak; 1c: eQTL + TF binding + matched TF motif + DNase peak; 1d: eQTL + TF binding + any motif + DNase peak; 1e: eQTL + TF binding + matched TF motif; 1f: eQTL + TF binding / DNase peak; 2a: TF binding + matched TF motif + matched DNase Footprint + DNase peak; 2b: TF binding + any motif + DNase Footprint + DNase peak; 2c: TF binding + matched TF motif + DNase peak; 3a: TF binding + any motif + DNase peak; 3b: TF binding + matched TF motif; 4: TF binding + DNase peak; 5: TF binding or DNase peak; 6: Motif hit; 7: Other.

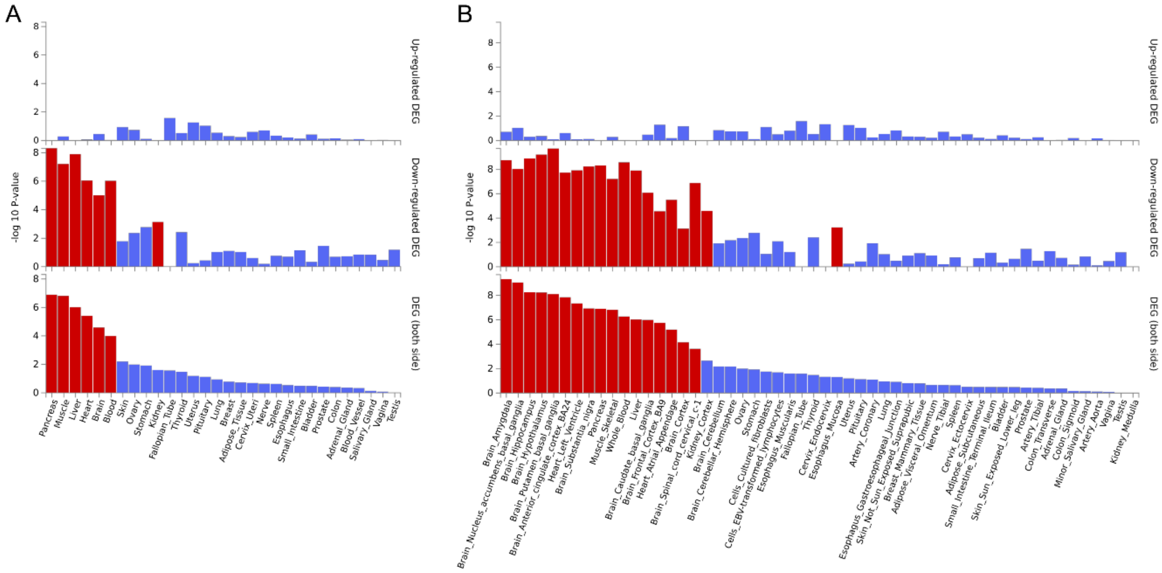

Supplementary Figure 5. GTEx enrichment analysis based on the 87 genes mapped by all strategies. (A) General tissue types. (B) Tissue types. The results have been exported from FUMA gene2func. P-values are two-tailed.

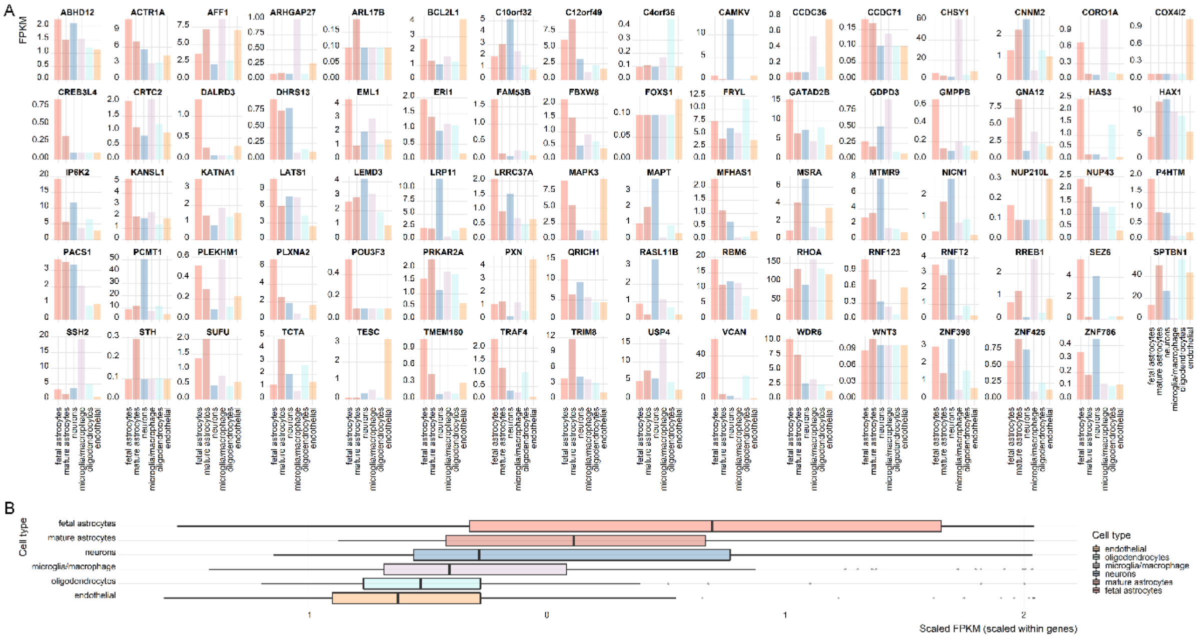

Supplementary Figure 6. Cell type analysis for the 87 common genes. Genes that were not expressed or that were missing in the data base were not included, yielding 79 genes. (A) Panel A shows profiles per gene. FPKM=Fragments Per Kilobase Million. (B) We scaled profiles within genes and compared them across genes (n=79), implicating fetal astrocytest with strongest overall expression. The lower and upper hinges of the boxplot correspond to the first and third quartiles, the center reflects the median. Pairwise t-test results (** indicates bonferroni significance): fetal astrocytes vs. mature astrocytes P=5.07e-03 | fetal astrocytes vs. neurons P=5.57e-04 ** | fetal astrocytes vs. oligodendrocytes P=4.53e-12 ** | fetal astrocytes vs. microglia/macrophage P=2.68e-06 ** | fetal astrocytes vs. endothelial P=4.49e-10 ** | mature astrocytes vs. neurons P=3.01e-01 | mature astrocytes vs. oligodendrocytes P=4.44e-08 ** | mature astrocytes vs. microglia/macrophage P=1.18e-02 | mature astrocytes vs. endothelial P=6.44e-06 ** | neurons vs. oligodendrocytes P=1.57e-04 ** | neurons vs. microglia/macrophage P=1.86e-01 | neurons vs. endothelial P=1.33e-03 ** | oligodendrocytes vs. microglia/macrophage P=1.42e-02 | oligodendrocytes vs. endothelial P=9.50e-01 | microglia/macrophage vs. endothelial P=4.41e-02.

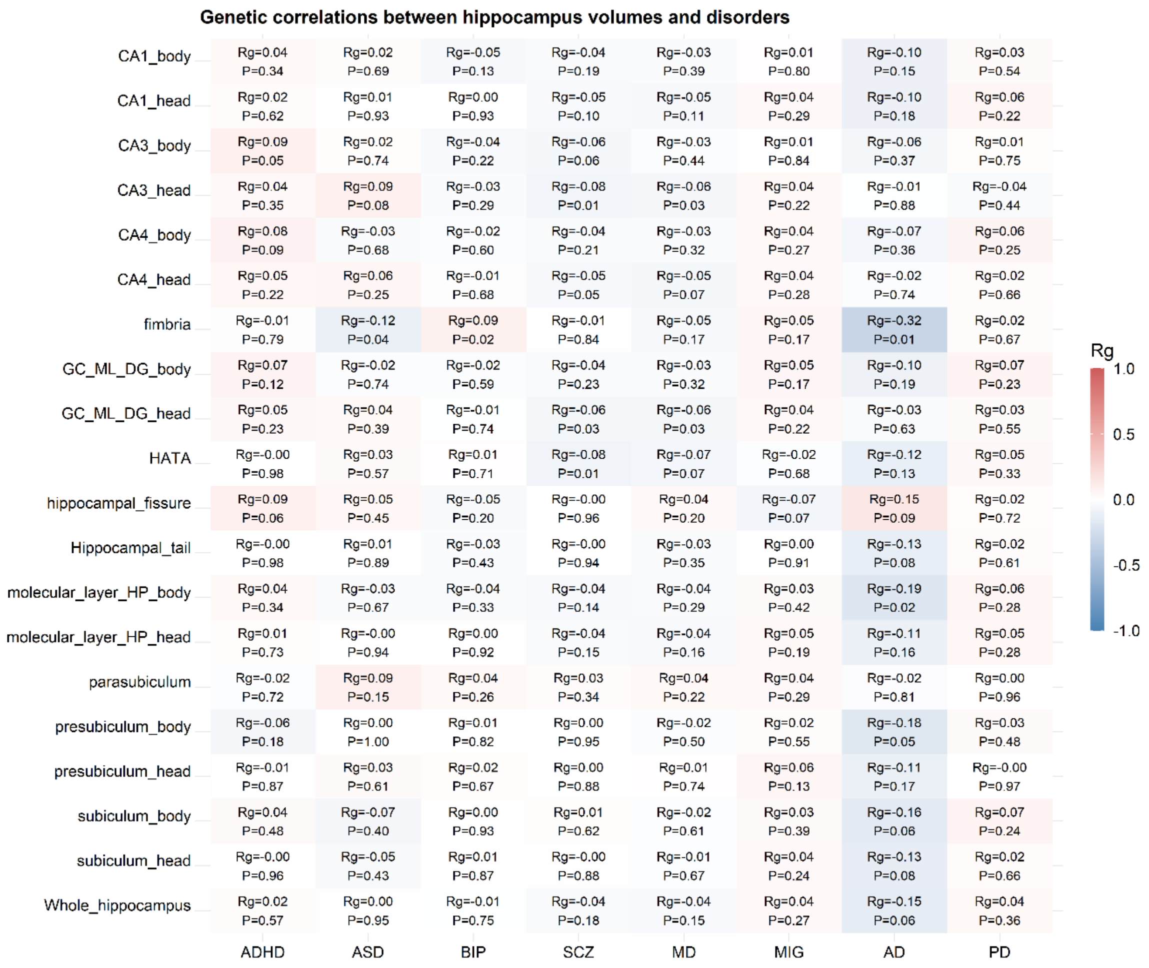

Supplementary Figure 7. LD-score regression based genetic correlations between hippocampus volumes and eight brain disorders. The analysis is based on the univariate statistics of the individual regions. Colors reflect correlation strengths. P-values are two-tailed.

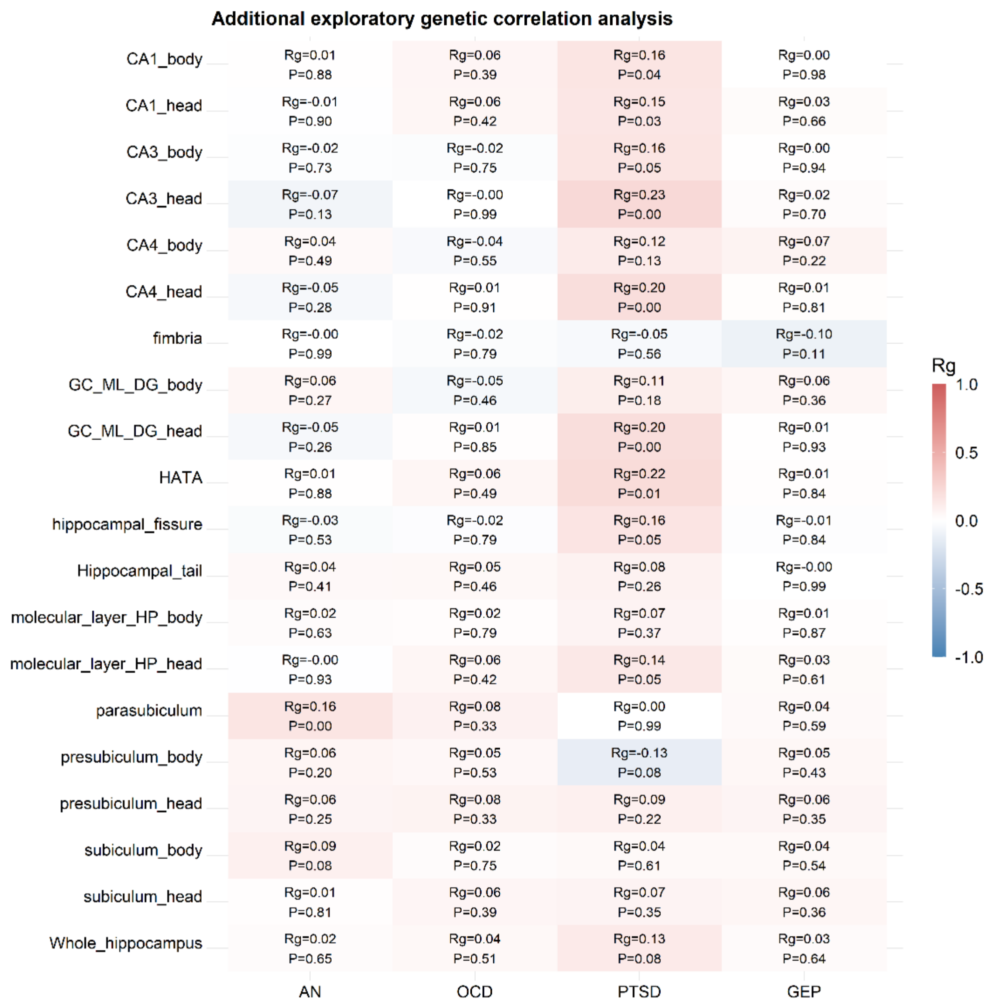

Supplementary Figure 8. Exploratory analysis, including additional disorders to test for genetic correlation with individual hippocampus regions. Colors reflect correlation strengths. P-values are two-tailed. Anorexia nervosa (AN[3]), obsessive-compulsive disorder (OCD[4]), posttraumatic stress disorder (PTSD[5]), and generalized epilepsy (GEP[6]). None of the correlations was significant when adjusting for the number of tests.

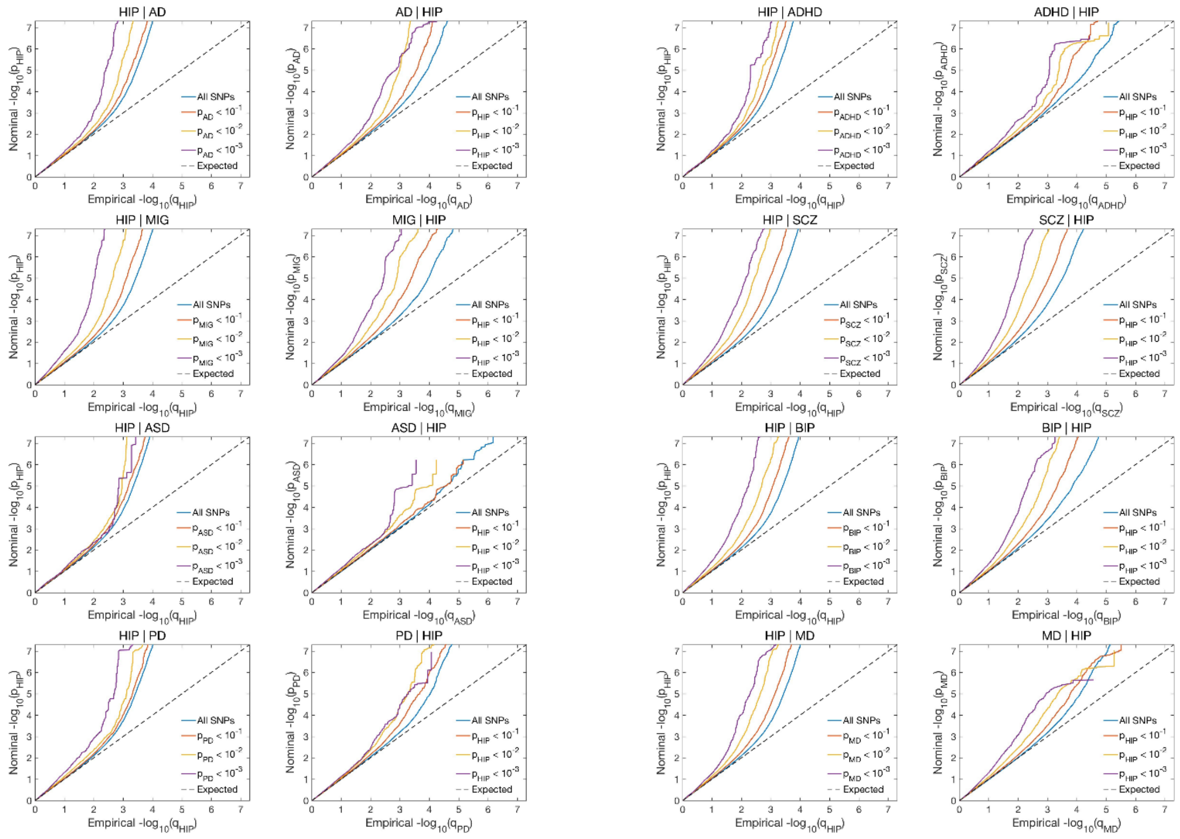

Supplementary Figure 9. Conditional Q-Q plots for hippocampus given associations with the disorder (left figures) and vice versa (right figures). ADHD: attention deficit hyperactivity disorder. AD: Alzheimer's disease. MIG: migraine. ASD: autism spectrum disorder. PD: Parkinson's disease. SCZ: schizophrenia. BIP: bipolar disorder. MD: major depression.

Supplementary References

1 van der Meer, D. *et al.* Brain scans from 21,297 individuals reveal the genetic architecture of hippocampal subfield volumes. *Mol Psychiatry* **25**, 3053-3065, doi:10.1038/s41380-018-0262-7 (2020).

2 Loughnan, R. J. *et al.* Generalization of Cortical MOSTest Genome-Wide Associations Within and Across Samples. *bioRxiv*, 2021.2004.2023.441215, doi:10.1101/2021.04.23.441215 (2021).

3 Watson, H. J. *et al.* Genome-wide association study identifies eight risk loci and implicates metabo-psychiatric origins for anorexia nervosa. *Nat Genet* **51**, 1207-1214, doi:10.1038/s41588-019-0439-2 (2019).

4 International Obsessive Compulsive Disorder Foundation Genetics, C. & Studies, O. C. D. C. G. A. Revealing the complex genetic architecture of obsessive-compulsive disorder using meta-analysis. *Mol Psychiatry* **23**, 1181-1188, doi:10.1038/mp.2017.154 (2018).

5 Nievergelt, C. M. *et al.* International meta-analysis of PTSD genome-wide association studies identifies sex- and ancestry-specific genetic risk loci. *Nat Commun* **10**, 4558, doi:10.1038/s41467-019-12576-w (2019).

6 International League Against Epilepsy Consortium on Complex, E. Genome-wide mega-analysis identifies 16 loci and highlights diverse biological mechanisms in the common epilepsies. *Nat Commun* **9**, 5269, doi:10.1038/s41467-018-07524-z (2018).
