# Supplementary material for: Distributed genetic architecture across the hippocampal formation implies common neuropathology across brain disorders
Source: Nat Commun. 2022 Jun 15;13:3436. doi: 10.1038/s41467-022-31086-w (PMC9200849; doi:10.1038/s41467-022-31086-w)
Supplement: Supplementary file 3 — Description of Additional Supplementary Files [file 41467_2022_31086_MOESM3_ESM.docx]

**Description of Additional Supplementary Files**

File Name: Supplementary Data 1

Description: LD-score regression based genetic correlations between hippocampus volumes (univariate analysis)

File Name: Supplementary Data 2

Description: Genetic loci associated with volumes of the hippocampal formation (multivariate GWAS; HIP)

File Name: Supplementary Data 3

Description: Functional annotation of single nucleotide polymorphisms (SNPs) in linkage disequilibrium (r2 ≥ 0.6) with one of the independent significant SNPs for HIP

File Name: Supplementary Data 4

Description: Mapping of significant HIP loci to genes

File Name: Supplementary Data 5

Description: Genome-wide gene-based association analyses for HIP

File Name: Supplementary Data 6

Description: Mapping of significant HIP loci to genes by all four strategies

File Name: Supplementary Data 7

Description: MAGMA Gene-Set Analysis for HIP

File Name: Supplementary Data 8A

Description: Gene Ontology gene-sets significantly associated with mapped genes for HIP

File Name: Supplementary Data 8B

Description: Gene Ontology gene-sets significantly associated with the 87 common mapped genes for HIP

File Name: Supplementary Data 9A

Description: Overrepresented pathways in mapped genes for HIP

File Name: Supplementary Data 9B

Description: Overrepresented pathways for the 87 common mapped genes for HIP

File Name: Supplementary Data 10

Description: LD-score regression based genetic correlations between hippocampus volumes and eight brain disorders (univariate analysis)

File Name: Supplementary Data 11

Description: Distinct genomic loci associated with Hippocampus (HIP) and migraine (MIG) at conjFDR<0.05

File Name: Supplementary Data 12

Description: Distinct genomic loci associated with Hippocampus (HIP) and parkinson (PD) at conjFDR<0.05

File Name: Supplementary Data 13

Description: Distinct genomic loci associated with Hippocampus (HIP) and alzheimer (AD) at conjFDR<0.05

File Name: Supplementary Data 14

Description: Distinct genomic loci associated with Hippocampus (HIP) and ADHD at conjFDR<0.05

File Name: Supplementary Data 15

Description: Distinct genomic loci associated with Hippocampus (HIP) and schizophrenia (SCZ) at conjFDR<0.05

File Name: Supplementary Data 16

Description: Distinct genomic loci associated with Hippocampus (HIP) and bipolar disorder (BIP) at conjFDR<0.05

File Name: Supplementary Data 17

Description: Distinct genomic loci associated with Hippocampus (HIP) and major depression (MD) at conjFDR<0.05

File Name: Supplementary Data 18

Description: Distinct genomic loci associated with Hippocampus (HIP) and Autism Spectrum Disorder (ASD) at conjFDR<0.05

File Name: Supplementary Data 19

Description: Genes mapped for each of the conjFDR<0.05 results for the different disorders
