# Supplementary material for: Distributed genetic architecture across the hippocampal formation implies common neuropathology across brain disorders
Source: Nat Commun. 2022 Jun 15;13:3436. doi: 10.1038/s41467-022-31086-w (PMC9200849; doi:10.1038/s41467-022-31086-w)
Supplement: Supplementary file 5 — Reporting Summary [file 41467_2022_31086_MOESM5_ESM.pdf]

nature portfolio

Corresponding author(s): Shahram Bahrami and Tobias Kaufmann

Last updated by author(s): Apr 27, 2022

Reporting Summary

Nature Portfolio wishes to improve the reproducibility of the work that we publish. This form provides structure for consistency and transparency in reporting. For further information on Nature Portfolio policies, see our Editorial Policies and the Editorial Policy Checklist.

Statistics

For all statistical analyses, confirm that the following items are present in the figure legend, table legend, main text, or Methods section.

n/a	Confirmed	
☐	☒	The exact sample size (*n*) for each experimental group/condition, given as a discrete number and unit of measurement
☐	☒	A statement on whether measurements were taken from distinct samples or whether the same sample was measured repeatedly
☐	☒	The statistical test(s) used AND whether they are one- or two-sided<br>*Only common tests should be described solely by name; describe more complex techniques in the Methods section.*
☐	☒	A description of all covariates tested
☐	☒	A description of any assumptions or corrections, such as tests of normality and adjustment for multiple comparisons
☐	☒	A full description of the statistical parameters including central tendency (e.g. means) or other basic estimates (e.g. regression coefficient) AND variation (e.g. standard deviation) or associated estimates of uncertainty (e.g. confidence intervals)
☐	☒	For null hypothesis testing, the test statistic (e.g. *F*, *t*, *r*) with confidence intervals, effect sizes, degrees of freedom and *P* value noted<br>*Give P values as exact values whenever suitable.*
☒	☐	For Bayesian analysis, information on the choice of priors and Markov chain Monte Carlo settings
☒	☐	For hierarchical and complex designs, identification of the appropriate level for tests and full reporting of outcomes
☐	☒	Estimates of effect sizes (e.g. Cohen's *d*, Pearson's *r*), indicating how they were calculated

Our web collection on statistics for biologists contains articles on many of the points above.

Software and code

Policy information about availability of computer code

Data collection	No software was used for data collection in this study as the data was aquired by the UK Biobank study team.
Data analysis	- The MRI data was analyzed using the standard recon-all pipeline in Freesurfer 5.3, and we subsequently segmented the hippocampal formation using Freesurfer 7.1<br>- We performed multivariate genome-wide association analysis using MOSTest available from https://github.com/precimed/mostest<br>- We performed conjunctional FDR analysis using pleioFDR available from https://github.com/precimed/pleiofdr/ (Matlab version 2017)<br>- We utilized the Functional Mapping and Annotation of GWAS (FUMA) platform available at https://fuma.ctglab.nl/ (version 1.3.7, incl MAGMA)<br>- We performed genetic correlation analysis using LD Score regression from https://github.com/bulik/ldsc/ (version 1.0)<br>- We estimated heritability using Genome-wide Complex Trait Analysis (GCTA)

For manuscripts utilizing custom algorithms or software that are central to the research but not yet described in published literature, software must be made available to editors and reviewers. We strongly encourage code deposition in a community repository (e.g. GitHub). See the Nature Portfolio guidelines for submitting code & software for further information.

Data

Policy information about availability of data

All manuscripts must include a data availability statement. This statement should provide the following information, where applicable:

- Accession codes, unique identifiers, or web links for publicly available datasets
- A description of any restrictions on data availability
- For clinical datasets or third party data, please ensure that the statement adheres to our policy

In this study we used brain imaging and genetics data from the UK Biobank [https://www.ukbiobank.ac.uk/], and GWAS summary statistics obtained from the Psychiatric Genomics Consortium [https://www.med.unc.edu/pgc/shared-methods/], 23andMe [https://www.23andme.com/], International headache genetics Consortium (IHGC) [http://www.headachegenetics.org/content/datasets-and-cohorts], the International Genomics of Alzheimer's Project [https://ctg.cncr.nl/software/summary_statistics], and the International Parkinson Disease Genomics Consortium [https://pdgenetics.org/resources]. The latter included 23andMe data, which was made available through 23andMe under an agreement with 23andMe that protects the privacy of the 23andMe participants [https://research.23andme.com/collaborate/#dataset-access/].
The summary statistics for hippocampal formation derived in this study is available in our github repository [https://github.com/norment/open-science]. FUMA results are available online [https://fuma.ctglab.nl/browse/371].

Field-specific reporting

Please select the one below that is the best fit for your research. If you are not sure, read the appropriate sections before making your selection.

☒ Life sciences ☐ Behavioural & social sciences ☐ Ecological, evolutionary & environmental sciences

For a reference copy of the document with all sections, see nature.com/documents/nr-reporting-summary-flat.pdf

Life sciences study design

All studies must disclose on these points even when the disclosure is negative.

Sample size	N=35,411 genotyped white British from the UK Biobank (age range: aged 45 – 82 years, mean: 64.4 years, sd: 7.5 years, 51.7% females) for the main analysis, and of N=5262 individuals with non-white ethnicity (age range: 45 - 81, mean: 62.9, sd: 7.6 years, 53.6% females) for the replication in independent data. No prior sample size estimation was conducted - we included all available data at the time the analysis was performed.
Data exclusions	None
Replication	We performed replication analysis in an independent sample of N=5262. To ensure that not only single locus associations replicate but that also the multivariate pattern of these associations are consistent in the discovery and replication sample, we implemented a multivariate replication procedure established in Loughnan et al. In brief, for each locus identified in the multivariate analysis in the discovery sample, this procedure derives a composite score from the mass-univariate z-statistics and tests for associations of the composite score with the genotype in the replication sample (for mathematical formulation see Loughnan et al. Four of the 177 loci could not be tested as the lead SNPs from the discovery sample were not available in the replication sample. For the remaining loci we report the percent of loci replicating at P<.05 and the percent of loci showing the same effect direction.<br>Attempts at replication were successful: We found that 68% of the loci replicated at P<.05 and 98% showed the same effect direction.
Randomization	Randomization is not applicable in the chosen study design. The group assignment for discovery or replication sample is based on ethnicity, and no group assignments that would require randomization are made.
Blinding	The study design did not require blinding (e.g. no case-control design).

Reporting for specific materials, systems and methods

We require information from authors about some types of materials, experimental systems and methods used in many studies. Here, indicate whether each material, system or method listed is relevant to your study. If you are not sure if a list item applies to your research, read the appropriate section before selecting a response.

Materials & experimental systems

n/a	Involved in the study	
☒	☐	Antibodies
☒	☐	Eukaryotic cell lines
☒	☐	Palaeontology and archaeology
☒	☐	Animals and other organisms
☐	☒	Human research participants
☒	☐	Clinical data
☒	☐	Dual use research of concern

Methods

n/a	Involved in the study	
☒	☐	ChIP-seq
☒	☐	Flow cytometry
☐	☒	MRI-based neuroimaging

Human research participants

Policy information about studies involving human research participants

Population characteristics	35,411 genotyped white British from the UK Biobank (age range: 45 – 82 years, mean: 64.4 years, sd: 7.5 years, 51.7% females). In addition, for replication, data on 5262 individuals with non-white ethnicity (age range: 45 - 81, mean: 62.9, sd: 7.6 years, 53.6% females).
Recruitment	We have not acquired new data and are not aware of recruitment biases likely to have a major impact on the results obtained in this study. Details on recruitment procedures can be found in the publications for the individual studies that contributed data (UK Biobank, PGC, etc).
Ethics oversight	The UK Biobank was approved by the North West Centre for Research Ethics Committee (11/NW/0382).

Note that full information on the approval of the study protocol must also be provided in the manuscript.

Magnetic resonance imaging

Experimental design

Design type	Anatomical MRI
Design specifications	See https://www.nature.com/articles/nn.4393
Behavioral performance measures	None

Acquisition

Imaging type(s)	Structural, T1w
Field strength	3T
Sequence & imaging parameters	TR=2000ms, TE=2.01ms, FA=8° (3 identical scanning sites), employing a Siemens 3T Skyra
Area of acquisition	Whole brain

Diffusion MRI ☐ Used ☒ Not used

Preprocessing

Preprocessing software	We processed T1-weighted images using the standard recon-all pipeline in Freesurfer 5.3, and subsequently segmented the hippocampal formation using Freesurfer 7.1.
Normalization	Standard procedures employed in Freesurfer (recon -all) were employed.
Normalization template	fsaverage
Noise and artifact removal	We used standard pipelines for anatomical data (Freesurfer recon -all).
Volume censoring	None

Statistical modeling & inference

Model type and settings	MOSTest, https://www.nature.com/articles/s41467-020-17368-1
Effect(s) tested	Multivariate genome-wide association and genetic overlap analysis, univariate genetic correlations

Specify type of analysis: ☐ Whole brain ☒ ROI-based ☐ Both

Anatomical location(s): Volumes of the hippocampal formation

Statistic type for inference (See Eklund et al. 2016): Permutation testing (MOSTest) and replication in independent data

Correction: The conjunctional FDR analysis uses FDR correction

Models & analysis

n/a	Involved in the study
☒	☐ Functional and/or effective connectivity
☒	☐ Graph analysis
☒	☐ Multivariate modeling or predictive analysis
